# Three-Dimensional Visualization of APEX2-Tagged Erg11 in Saccharomyces cerevisiae Using Focused Ion Beam Scanning Electron Microscopy

**DOI:** 10.1128/mSphere.00981-19

**Published:** 2020-02-05

**Authors:** Winnie Kerstens, Anneke Kremer, Michelle Holtappels, Peter Borghgraef, Saskia Lippens, Patrick Van Dijck

**Affiliations:** a VIB-KU Leuven Center for Microbiology, Flanders, Belgium; b Laboratory of Molecular Cell Biology, Institute of Botany and Microbiology, KU Leuven, Leuven, Belgium; c VIB Bioimaging Core, VIB, Ghent, Belgium; d Department of Biomedical Molecular Biology, Ghent University, Ghent, Belgium; University of Georgia

**Keywords:** FIB-SEM, electron microscopy, *Saccharomyces cerevisiae*, yeast, APEX2, DAB, protein localization, spheroplast

## Abstract

The determination of the exact location of a protein in the cell is essential to the understanding of biological processes. Here, we report for the first time the visualization of a protein of interest in Saccharomyces cerevisiae using focused ion beam scanning electron microscopy (FIB-SEM). As a proof of concept, the integral endoplasmic reticulum (ER) membrane protein Erg11 has been C-terminally tagged with APEX2, which is an engineered peroxidase that catalyzes an electron-dense deposition of 3,3′-diaminobenzidine (DAB), as such marking the location of the fused protein of interest in electron microscopic images. As DAB is unable to cross the yeast cell wall to react with APEX2, cell walls have been partly removed by the formation of spheroplasts. This has resulted in a clear electron-dense ER signal for the Erg11 protein using FIB-SEM. With this study, we have validated the use of the APEX2 tag for visualization of yeast proteins in electron microscopy. Furthermore, we have introduced a methodology that enables precise and three-dimensional (3D) localization studies in yeast, with nanometer resolution and without the need for antibody staining. Because of these properties, the described technique can offer valuable information on the molecular functions of studied proteins.

**IMPORTANCE** With this study, we have validated the use of the APEX2 tag to define the localization of proteins in the model yeast S. cerevisiae. As such, FIB-SEM can identify the exact 3D location of a protein of interest in the cell with nanometer-scale resolution. Such detailed imaging could provide essential information on the elucidation of various biological processes. APEX2, which adds electron density to a fused protein of interest upon addition of the substrate DAB, originally was used in mammalian studies. With this study, we expand its use to protein localization studies in one of the most important models in molecular biology.

## Abstract (precis)

With this study, we have validated the use of the APEX2 tag to define the localization of proteins in the model yeast S. cerevisiae. As such, FIB-SEM can identify the exact 3D location of a protein of interest in the cell with nanometer-scale resolution. Such detailed imaging could provide essential information on the elucidation of various biological processes. APEX2, which adds electron density to a fused protein of interest upon addition of the substrate DAB, originally was used in mammalian studies. With this study, we expand its use to protein localization studies in one of the most important models in molecular biology.

## INTRODUCTION

The identification of the subcellular localization of a protein is important to define its molecular function. Electron microscopy (EM) techniques have a prominent role, because they have the advantage of resolution superior to that of light microscopy techniques and further allow the visualization of the complete cellular context. Several technologies can be used to provide EM information in three dimensions (3D), also referred to as volume-EM. Since the turn of the century, serial block-face scanning electron microscopy (SBF-SEM) and focused ion beam scanning electron microscopy (FIB-SEM) have been introduced for volume-EM on biological samples (1, 2). SBF-SEM and FIB-SEM both represent a serial block-face imaging technique, meaning that the surface of a sample block is successively sectioned and imaged. In SBF-SEM, the top layer of a given sample (usually 50 to 70 nm) is removed with a diamond knife ultramicrotome inside the SEM vacuum chamber, followed by consecutive imaging of the block-face with the SEM. FIB-SEM, on the other hand, depends on the use of two beams: a focused ion beam for sectioning, typically gallium ions, and an electron beam for imaging. Previously mainly used in material sciences, the FIB mills sections as thin as five nanometers from the surface, while the SEM produces nanometer-resolution images, resulting in image stacks that allow for volume reconstructions at isotropic voxels in the nanometer range. Segmentation of these isotropic data sets provides precise and high-resolution 3D visualization of organelles or structures of interest (1–3). Compared to 3D transmission electron microscopy (TEM) techniques, such as serial section TEM and electron tomography, FIB-SEM imaging represents a fully automated process of sample sectioning by the FIB and detection by the SEM, making it a far less labor-intensive 3D imaging technique. However, imaging by TEM could offer higher-resolution results than SEM (2, 4).

The total volume that can be imaged by FIB-SEM is limited, but volumes of 20 μm in all directions are standard. This means that the volume of a whole yeast cell can be covered easily with this technique (4). One of the strengths of EM techniques is that they provide a comprehensive view of a sample, because the detected electron-dense signal comes from heavy-metal stainings that bind to lipid membranes without selectivity for a specific subcellular structure. At the same time, this is also a weakness when one tries to use EM for specific localization studies, e.g., the determination of protein localization signals in the cell. This has resulted in the need for electron-dense tags that render a clear above-background signal in EM, similar to fluorescent protein tags that are used in light microscopy techniques. Several tools, e.g., miniSOGs, FlAsh/ReAsh, and quantum dots, have been developed to generate electron-dense signals at the location of interest in an EM-prepared sample.

Here, we report for the first time the use of an APEX2 tag for EM in the yeast S. cerevisiae, and we have made use of FIB-SEM imaging to determine the exact location of the fused protein of interest in 3D. APEX2 is an engineered peroxidase that catalyzes the H_2_O_2_-dependent polymerization of 3,3′-diaminobenzidine (DAB) into localized precipitates, which recruit osmium and create an electron-dense signal for EM imaging (5). The tag has been used in the context of EM imaging in human cells and zebrafish (6–8). We have optimized the sample preparation to detect the APEX2-based signal in yeast cells by preparing spheroplasts, which enhances the uptake of DAB into the cells. As such, we have validated the use of APEX2 as an electron-densifying tag for FIB-SEM of yeast samples.

The APEX2 sequence has been added as a C-terminal tag to the endoplasmic reticulum (ER) membrane protein Erg11. Lanosterol 14α-demethylase, or Erg11, is encoded by the essential *ERG11* gene in S. cerevisiae (9). The protein belongs to the cytochrome P450 (CYP) superfamily, which comprises a large group of monooxygenases that can be found in all biological kingdoms. They share some specific characteristics, such as a prosthetic heme group (10). Therefore, Erg11 is also known as CYP51. CYPs can be found as integral ER membrane or mitochondrial inner membrane proteins in eukaryotes (11). S. cerevisiae Erg11 localizes to the ER membrane (12, 13). It catalyzes a crucial step in the biosynthesis pathway of ergosterol by the conversion of lanosterol to 4,4-dimethylergosta-8,14,24-trienol. Sterols carry structural and regulatory functions that are of vital importance to the cell, e.g., to membrane permeability, to the activity of membrane-bound proteins, and to the cellular growth rate (9). In yeast, ergosterol is the main sterol incorporated in membranes, similar to cholesterol in mammals. Because of its function in sterol production, Erg11 is a well-characterized protein (9, 13, 14). Moreover, Erg11 is the target of the azole class of antifungals, and upregulation of the expression of *ERG11* is a major cause of clinical azole-resistant isolates, underscoring the importance of Erg11 in yeast biology (15, 16).

## RESULTS AND DISCUSSION

### The APEX2 tag is functional in Saccharomyces cerevisiae and does not interfere with the essential function of Erg11 when fused to its C terminus.

**FIG 1:**
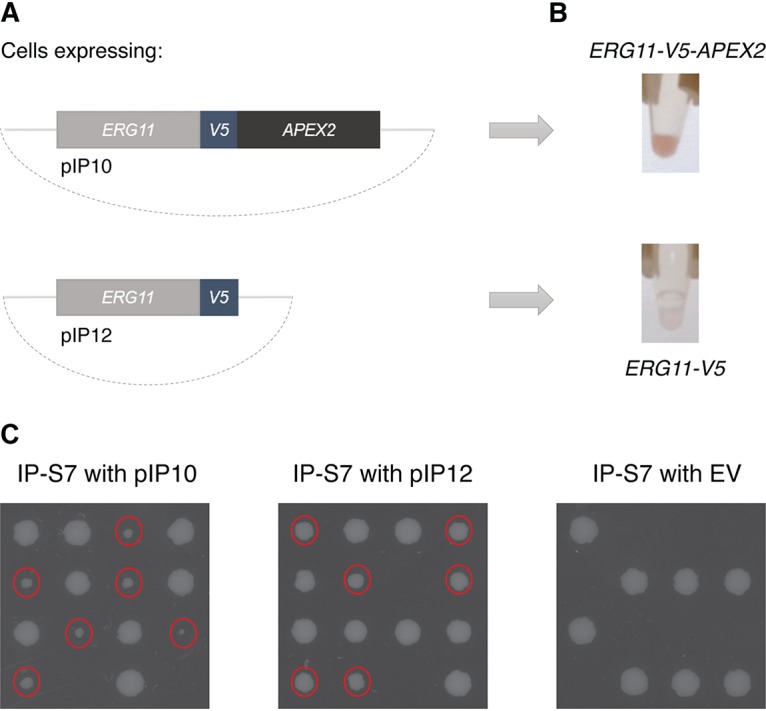
APEX2 is a functional tag in S. cerevisiae. (A) Two plasmids were constructed, expressing APEX2-tagged *ERG11-V5* or the control construct *ERG11-V5*. The plasmids were transformed into S. cerevisiae. (B) After overnight growth of the yeast cells in –Leu medium, they were lysed and the proteins were extracted. The protein extracts then were incubated with a DAB solution with H_2_O_2_ to detect whether the APEX2 tag fused to Erg11 is expressed and active. Indeed, for the cells expressing *ERG11-V5-APEX2*, the protein extract was colored brown, indicating that the APEX2 tag retained its peroxidase activity and that the chimeric protein is functionally expressed in S. cerevisiae. As expected, the protein extract of the control cells remained white. (C) Tetrad dissection of IP-S7 cells transformed with the pIP10 plasmid containing *ERG11-V5-APEX2* or the pIP12 plasmid containing *ERG11-V5,* or the empty vector (EV) pBEVY-L as a negative control, shows that the Erg11 proteins expressed from the plasmids are functional, as they can sustain the germination of spores lacking endogenous *ERG11*. The smaller colonies (indicated by red dots) were shown to lack endogenous *ERG11* by PCR. Replating these cells on selective −Leu medium showed that all the smaller colonies retained the pIP10 or pIP12 plasmid.

Two constructs have been generated and expressed in S. cerevisiae from the strong glyceraldehyde-3-phosphate dehydrogenase (GPD) promoter on the pBEVY-L plasmid, expressing either the *APEX2* sequence C-terminally to *V5*-tagged *ERG11* (pIP10) or the *ERG11-V5* construct without the APEX2 tag (pIP12) as a negative control (Fig. 1A). To test whether the Erg11-APEX2 chimeric protein is successfully expressed and whether the APEX2 tag maintains its peroxidase function in yeast cells, lysates of the S. cerevisiae control cells and *ERG11-V5-APEX2*-expressing cells were incubated with DAB and H_2_O_2_. Active APEX2 peroxidase will convert the DAB substrate into a brown precipitate. Indeed, as seen from Fig. 1B, the protein extracts of *ERG11-V5-APEX2*-expressing cells obtained a brown color in this descriptive experiment, while the extracts of the control cells overexpressing *ERG11-V5* remained colorless, indicating that the *ERG11-V5-APEX2* construct is expressed and that APEX2 is functional in S. cerevisiae. This agrees with previous studies, where the functionality of the APEX2 protein has been demonstrated in S. cerevisiae for the analysis of protein-protein interactions (5, 17).

Furthermore, we have confirmed that the APEX2 tag does not block the essential function of Erg11. To examine whether the C-terminal tag affects the function of the Erg11 protein, we have transformed the heterozygous strain IP-S7, which lacks one of the two wild-type *ERG11* alleles, with the pIP10 and pIP12 plasmids. As a negative control, we transformed IP-S7 with the empty vector pBEVY-L. As *ERG11* carries an essential function, sporulation and tetrad dissection of the IP-S7 diploid strain would result in germination of only those spores carrying the wild-type *ERG11* allele. However, when the IP-S7 strain is transformed with the pIP10 and pIP12 plasmids, all four spores should germinate if the *ERG11-V5-APEX2* and *ERG11-V5* constructs are functional. The tetrads resulting from the sporulation of IP-S7 transformed with the empty vector, on the other hand, should not germinate when the endogenous *ERG11* allele is not present, as then no functional Erg11 protein is expressed. The results of this experiment are shown in Fig. 1C. Indeed, spores lacking endogenous *ERG11* and expressing the pIP10 or pIP12 plasmid were able to germinate, while spores with the empty vector did not germinate when the endogenous *ERG11* allele was absent. The presence of endogenous *ERG11* was checked by PCR, while the presence of the plasmids after sporulation and tetrad dissection was confirmed by replating the tetrads on selective medium. This experiment confirms that the Erg11 protein is functional when it is fused to the APEX2 tag. As can be seen from Fig. 1C, the spores expressing solely *ERG11-V5-APEX2* grow slower than the spores with the wild-type *ERG11* allele, which is most likely ascribed to the APEX2 tag, as spores with only *ERG11-V5* grow at a rate more similar to that of the wild-type spores.

### Spheroplast formation does not interfere with FIB-SEM sample preparation.

**FIG 2:**
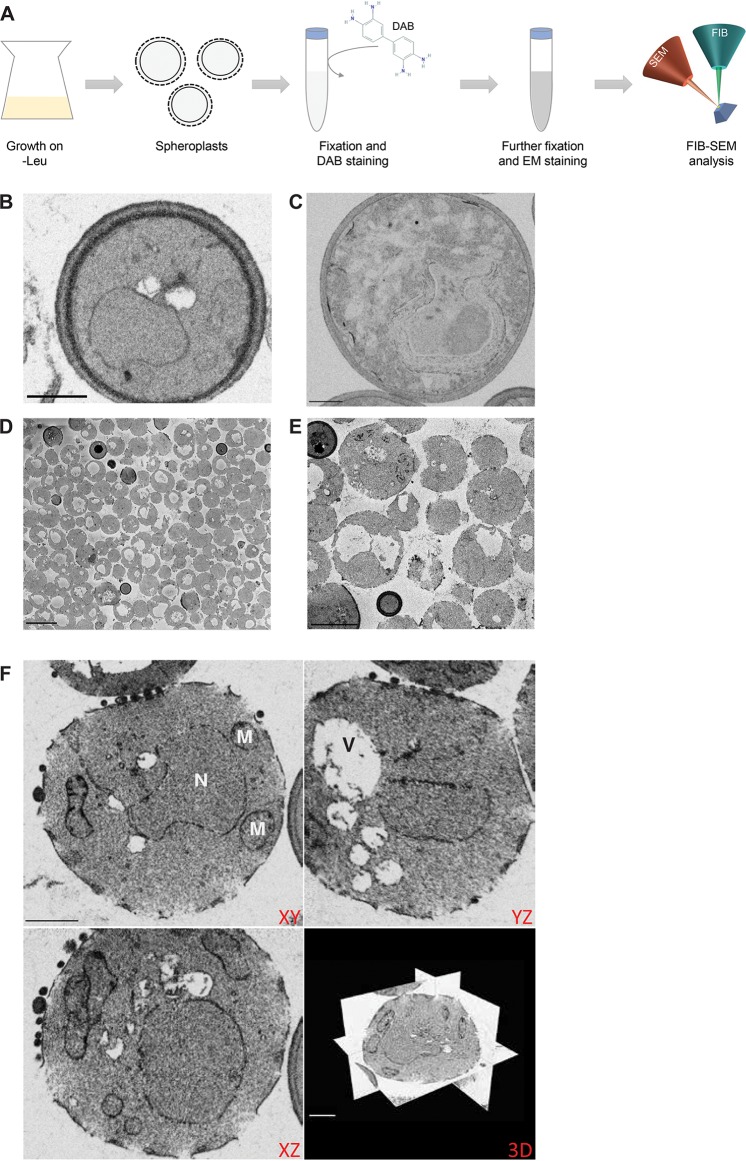
Spheroplast formation does not interfere with FIB-SEM sample preparation. (A) The workflow of the experiment is presented, where cells are grown overnight in −Leu medium and subsequently subjected to spheroplast formation. The spheroplasts next are fixed, incubated with DAB (structure retrieved from the PubChem Database, CID = 7071; https://pubchem.ncbi.nlm.nih.gov/compound/7071), and further prepared for FIB-SEM analysis. (B and C) FIB-SEM images of nonspheroplast control cells expressing Erg11-V5 without the APEX2 tag (B) and nonspheroplast cells expressing Erg11-V5-APEX2 (C) show low electron-dense signal in both samples (scale bars, 1 μm). (D and E) Representative images of SBF-SEM data of control cells after spheroplast formation, showing that spheroplast formation was successful for most cells (D) and organelle ultrastructure was preserved (E) (scale bars, 5 μm). (F) Orthogonal views of FIB-SEM data of the same sample of control cells confirm the preservation of organelle ultrastructure and cell shape (scale bars, 1 μm). N, nucleus; M, mitochondria; V, vacuole.

Cells expressing the control Erg11-V5 construct or APEX2-tagged Erg11-V5 were incubated with DAB and prepared for FIB-SEM imaging, as depicted in the workflow presented in Fig. 2A. Compared to the control cells (Fig. 2B), there was no clear enrichment of electron-dense signal in the images of *ERG11-V5-APEX2*-expressing cells (Fig. 2C), although we have shown that APEX2 is functional in these cells (Fig. 1B). The absence of electron-dense signal could be caused by failure of DAB to penetrate the yeast cell wall. To test this, we generated spheroplasts, which lack a great part of their cell wall.

Spheroplast formation is a frequently used technique in yeast research. It is based on two important factors: a cell wall-digesting enzyme, lyticase in our case, directed at the cell wall glucans, and a reducing factor, dithiothreitol (DTT) (18, 19). Although the lyticase is washed away after the preparation of the spheroplasts, it is important to check the integrity of the studied protein after spheroplast formation if the described method is used for the study of glycoproteins or related proteins, e.g., through an enzymatic assay. Furthermore, it should be ensured that the subcellular localization of the protein is not altered due to the possible loss of the oligosaccharide part. The reducing agent also could affect the subcellular localization or stability of proteins. In our protocol, the cells were first treated with DTT and thereafter incubated with lyticase. As such, the reducing agent has been largely removed prior to the moment where the cell wall is disrupted and spheroplasts are formed. Nonetheless, it should be tested whether the reducing agent has an influence on the protein under study. The S. cerevisiae Erg11 protein carries no known intramolecular disulfide bonds.

The quality of the spheroplasts was first assessed by SBF-SEM imaging of a sample of control cells. The advantage of SBF-SEM is that we can image larger volumes than those for FIB-SEM, meaning that in a short run (300 sections of 70 nm) a large sample of yeast cells can be imaged and checked for spheroplast formation and integrity of organelle ultrastructure. Figure 2D and E indicates that the degradation of the cell wall was successful in most of the yeast cells in these samples (Fig. 2D) and that cell organelles can still be clearly distinguished (Fig. 2E). Subsequent FIB-SEM imaging of the same control sample shows that the overall structure of the cells indeed has not been affected by spheroplast formation and that this process has no effect on FIB-SEM imaging and sectioning (Fig. 2F).

### FIB-SEM analysis of APEX2-tagged Erg11 in Saccharomyces cerevisiae spheroplasts.

**FIG 3:**
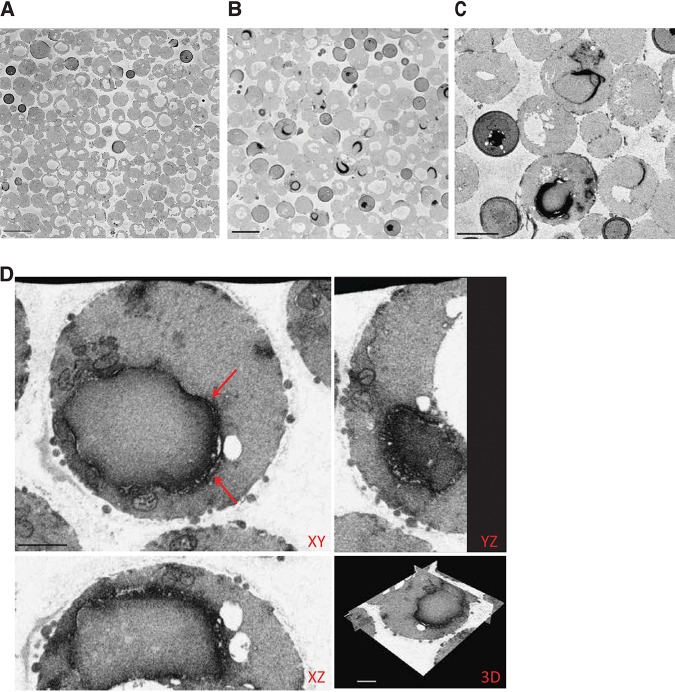
SBF-SEM and FIB-SEM analysis of Erg11-APEX2 in S. cerevisiae. (A, B, and C) Visualization of multiple cells by SBF-SEM after spheroplast formation shows that compared to control spheroplast cells (A), the APEX2-tagged Erg11 electron-dense signal can be clearly observed at the ER in several cells (B and C) (scale bars, 5 μm). (D) Orthogonal views of FIB-SEM data of spheroplasts expressing Erg11-V5-APEX2 show clear localization of the Erg11 protein to the ER (scale bars, 1 μm). Aberrant ER membrane structures are indicated by red arrows.

After validating that spheroplast formation does not interfere with FIB-SEM analysis, S. cerevisiae spheroplasts expressing APEX2-tagged Erg11 were incubated with DAB and prepared for volume-EM. To obtain a general overview of the electron-dense signal and test the consistent expression of APEX2-tagged Erg11 in the spheroplasts, the sample again was first imaged by SBF-SEM and compared to the SBF-SEM data of the control cells (Fig. 3A and 2D and E). SBF-SEM imaging of the spheroplasts expressing the chimeric Erg11-V5-APEX2 protein clearly shows the enrichment of electron-dense signal at the ER (Fig. 3B and C; see also Movie S1 in the supplemental material). Based on this SBF-SEM data for the Erg11-V5-APEX2 sample, we have tried to quantify the amount of spheroplast cells and the success rate of DAB staining. Unfortunately, the vacuoles of the spheroplasts have the same intensity as that of the background and the vacuoles have dimensions similar to those of the intercellular regions, making it very difficult to use software for quantification purposes. Therefore, we have manually counted the total number of cells in the SBF-SEM data set. Out of a total of 468 cells, 329, or 70.3%, were identified as proper spheroplast cells. Seventy-three of those spheroplasts had a clear electron-dense staining of the ER. Therefore, the estimated success rate of DAB staining for the *ERG11-V5-APEX2* spheroplasts is 22.2%. These results indicate that the APEX2 visualization tag in yeast also could be applied in other EM techniques. As several spheroplasts showed a clear electron-dense staining, the cells were further imaged at high resolution with the FIB-SEM.

Spheroplast cells expressing the Erg11-V5-APEX2 protein generally show a clear electron-dense signal in the ER (Fig. 3D). The Erg11 signal is detected in cortical but mainly in perinuclear ER. Moreover, some aberrant ER membrane structures can be observed (indicated by the arrows). For several CYPs, it has been shown that their overexpression induces ER stress, as the ER needs to accommodate the increased synthesis rates of the proteins (20). Aberrant ER membrane structures can be observed as stacks of paired ER membranes abundantly shaped around the nucleus, which have been termed karmellae, or as concentric membrane structures throughout the cytoplasm, termed whorls (20, 21). The multilayered ER membranes can be detected both in control samples and in the Erg11-APEX2 sample but are most easily detected in the latter (Fig. 3D and Fig. S1A versus E) because of the higher electron-dense signal of Erg11-APEX2. This is even more emphasized when using intensity-based thresholding (Fig. S1B and C) and displaying only a range of darker pixels/voxels. The result of the thresholding can be used to generate a 3D rendering of the structure of interest, here showing the multilayered ER surrounding the nucleus (Fig. S1D).

10.1128/mSphere.00981-19.1 FIG S1 Electron-dense Erg11-APEX2 ER signal shows multilayered ER and can be used for segmentation. (A) FIB-SEM data from cells expressing *ERG11-V5-APEX2* show multilayered ER membranes (scale bar, 1 μm). (B) Using Fiji software, a threshold was set on the pixel intensity (in red), allowing us to segment the highest intensities, which are APEX2-labeled ER structures, from the FIB-SEM dataset. (C) The result from this thresholding shows only APEX2-labeled ER, which now can be used for 3D visualization (scale bar, 1 μm). (D) Imaris software was used to render the thresholded FIB-SEM data in 3D, showing the multilayered ER surrounding the nucleus (scale bar, 500 nm). (E) FIB-SEM data of cells expressing the *ERG11-V5* control also show multilayered ER membranes, indicated by the arrows (scale bar, 1 μm). Download FIG S1, PDF file, 1.0 MB. Copyright © 2020 Kerstens et al. 2020 Kerstens et al. This content is distributed under the terms of the Creative Commons Attribution 4.0 International license.

### Conclusions.

So far, the APEX2 tag has already been used in yeast experiments for covalently linking proteins adjacent to the APEX2-fused protein of interest to biotin, so that these proteins can be affinity purified and identified through mass spectrometric analysis (17). Here, we show the use of APEX2 as a protein tag in S. cerevisiae for visualization and localization of a protein of interest with EM, confirming previous studies for APEX2-based protein localization in mammalian cells. We have optimized a 3D-EM FIB-SEM protocol, using spheroplasts and APEX2-mediated DAB conversion for electron-dense staining. The generation of spheroplasts for FIB-SEM imaging overall does not interfere with the general intracellular morphology of the yeast cells. As such, we were able to determine the localization of the Erg11 protein in the ER and observed effects of its overexpression on the general ER structure.

## MATERIALS AND METHODS

### Strains and culture media.

The strains that we used are listed in Table 1. Cells were grown in −Leu dropout medium consisting of 0.069% CSM-Leu (MP Biomedicals), 0.17% Difco yeast nitrogen base without amino acids and ammonium sulfate, 0.5% ammonium sulfate, and 2% glucose at pH 5.5. Media were solidified by adding 1.5% agar, and the pH was buffered to 6.5. Cells were incubated at 30°C throughout the experiments, except for the tetrad dissection experiment, where the diploids were induced to sporulate on sporulation medium (1% potassium acetate KAc, 0.05% KHCO_3_, 1.5% agar, adjusted to pH 6 by HCl) at 23°C. Before sporulation, the diploids were grown in presporulation medium (0.3% peptone, 0.8% yeast extract, and 2% potassium acetate). Tetrads were dissected on YPD medium (1% yeast extract, 2% Bacto peptone, 2% glucose, and 1.5% agar).

**TABLE 1:** Strain and plasmid list and the APEX2 sequence used in the constructs

Strain, plasmid, or peroxidase	Genotype, description, or sequence	Vector	Reference or source
Strains			
AFC202	Wild-type BY4742 with *ERG11*::*ERG11-3xHA*		23
IP-S7	Wild-type BY4743 with *ERG11/ERG11*::*3xHA*		This study
Plasmids			
pBEVY-L	Empty vector		24
pIP9	APEX2	pBEVY-L	This study
pIP10	*GPD1pro*::*ERG11-V5-APEX2*	pBEVY-L	This study
pIP12	*GPD1pro*::*ERG11-V5*	pBEVY-L	This study
Peroxidase			
APEX2	GCATCTAGAGCAGGAAAGTCTTACCCAACTGTGAGTGCTGATTACCAGGA CGCCGTTGAGAAGGCGAAGAAGAAGCTCAGAGGCTTCATCGCTGAGAAGA GATGCGCTCCTCTAATGCTCCGTTTGGCATTCCACTCTGCTGGAACCTTT GACAAGGGCACGAAGACCGGTGGACCCTTCGGAACCATCAAGCAC		

### Designing APEX2 constructs for Saccharomyces cerevisiae.

For FIB-SEM imaging, *ERG11-V5* was ligated into BamHI-XbaI-cut pBEVY-L. APEX2 with XbaI and PstI restriction sites was ordered as a gBlock (Integrated DNA Technologies) and ligated into pBEVY-L to generate pIP9 (Table 1). APEX2 next was PCR amplified with primers C-22 (GGACTTGATTCAACAACTAGTGCAGCAGGAAAGTCTTACCCAACTGT) and C-23 (GAAGTCCAAAGCTTGCATGCCTGCAGGTCGACTCTAGATTAGGCATCAGCAAACCCAAG) from pIP9 and ligated to the 3′ end of the *ERG11-V5* construct in pBEVY-L that was cut with SalI-PstI, generating the *ERG11-V5-APEX2* plasmid using NEBuilder (pIP10) (Table 1). The control construct pIP12 with *ERG11-V5* lacking the APEX2 tag was constructed by ligating *ERG11-V5* into BamHI-XbaI-cut pBEVY-L with NEBuilder. The ligations were transformed to Escherichia coli TOP10, and the respective plasmids were subsequently transformed to AFC202 S. cerevisiae cells.

### Protein extraction and DAB staining.

An overnight preculture was diluted to an optical density at 600 nm (OD_600_) of 0.2. After 16 h of growth, cells were harvested. The protocol of Diekert et al. (22) was used for crude membrane protein extraction. Briefly, cells were washed once in lysis buffer (0.6 M sorbitol, 20 mM HEPES-KOH [pH 7.4], and cOmplete EDTA-free protease inhibitor [Roche]), after which 1% Triton X-100 was added to the lysis buffer. Subsequently, the cells were broken in a FastPrep machine (MP Biomedicals) by glass beads. The samples next were centrifuged for 5 min at 3,000 rpm to separate the supernatant containing the protein fraction from the cell debris. The lysis step was repeated once. The isolated supernatant then was incubated with a 0.054% DAB-HCl solution, to which 1:1,000 of 30% H_2_O_2_ was added for detection of APEX2 activity. This experiment was repeated more than three times.

### Tetrad dissection.

After growing overnight in presporulation medium, the diploid strain IP-S7 was induced to sporulate on sporulation medium at 23°C. Tetrads were dissected with a micromanipulator (Singer Instruments) on YPD medium. Results are shown after 4 days of growth. PCR analysis confirmed that the smaller germinating colonies lacked the endogenous *ERG11* allele, while replating them on CSM-Leu medium, which is selective for the pBEVY-L based plasmids, confirmed that the smaller germinating colonies contained the *ERG11-V5-APEX2* or *ERG11-V5* plasmid.

### Spheroplast formation for FIB-SEM imaging.

Precultures were grown in −Leu dropout medium. Cells then were diluted to an OD of 0.2 and grown overnight. Spheroplasts were prepared by incubating the cells in a 0.1 M Tris-SO_4_–10 mM DTT solution for 20 min at 30°C with shaking at 200 rpm. The cells were resuspended in sorbitol buffer (1.2 M sorbitol and 20 mM KPi at pH 7.4), and 0.25 mg of lyticase was added per gram of wet weight. The cells were incubated at 30°C for 30 min to 60 min, with shaking at 200 rpm, until about 70% to 80% of the cells had become spheroplasts, as measured by spectrophotometric analysis. The cells were harvested and washed in sorbitol buffer.

### Fixation of the Saccharomyces cerevisiae cells, DAB staining, and sample preparation for FIB-SEM imaging.

A 1:1 ratio of spheroplasts and fixative 1 (3% paraformaldehyde [PFA; EMS] and 6% glutaraldehyde [GA; EMS] in 0.05 M sodium cacodylate [pH 7.2]) was incubated on ice for ten minutes. The pelleted spheroplasts next were carefully resuspended in fixative 2 (1.5% PFA and 3% GA in 0.05 M sodium cacodylate) at a 1:1 ratio and incubated overnight at 4°C. The next day, the fixative was removed by washing with 0.05 M sodium cacodylate. The samples were stained for 20 min in a 0.054% DAB-HCl solution with 1:1,000 of 30% H_2_O_2_ to obtain an electron-dense staining of the APEX2 tag that was fused to the *ERG11* constructs. The staining was stopped by washing the spheroplasts in 0.1 M sodium cacodylate. To protect the spheroplasts during the following EM preparation steps, a pellet of yeast cells was embedded in 2% low-melting-point agarose (Sigma) in 0.05 M sodium cacodylate. After an additional washing step, the pellets were incubated in 6% potassium permanganate for 1 h and again washed in 0.05 M sodium cacodylate. Samples next were dehydrated using solutions of increasing ethanol (EtOH) concentration for 15 min each (7%, 30%, 50%, and 70% [once each] and 100% EtOH [twice]). After the 50% EtOH step, samples were additionally incubated in 1% OsO_4_ (EMS), 50% EtOH for 1 h. Subsequent resin embedding was done using low-viscosity Spurr’s solution (EMS). Embedded cells were mounted on aluminum SEM stubs (diameter, 12 mm), and samples were coated with or without 20 nm of platinum (Quorum Q150T ES). FIB-SEM imaging was performed using a Zeiss Crossbeam 540 system with Atlas5 software. The FIB was set to remove 5-nm sections by propelling gallium ions at the surface (probe current, 700 pA). Imaging was done at 1.5 kV and 600 pA using an EsB (energy-selective backscattered) detector, with the EsB grid set at −1,200 V. Registration of the resulting data set was done using IMOD (http://bio3d.colorado.edu/imod/). Fiji (https://fiji.sc/) was used for thresholding and orthogonal views, and 3D renderings were done in Imaris (Bitplane).

### Data availability.

All data are available from the authors.

10.1128/mSphere.00981-19.2 MOVIE S1 This movie is a compilation of all individual SBF-SEM images of the Erg11-V5-APEX2 spheroplast sample placed one after the other. Download Movie S1, AVI file, 19.1 MB. Copyright © 2020 Kerstens et al. 2020 Kerstens et al. This content is distributed under the terms of the Creative Commons Attribution 4.0 International license.
