# Supplementary figures and images for: Three-Dimensional Visualization of APEX2-Tagged Erg11 in Saccharomyces cerevisiae Using Focused Ion Beam Scanning Electron Microscopy
Source: mSphere. 2020 Feb 5;5(1):e00981-19. doi: 10.1128/mSphere.00981-19 (PMC7002314; doi:10.1128/mSphere.00981-19)

**A**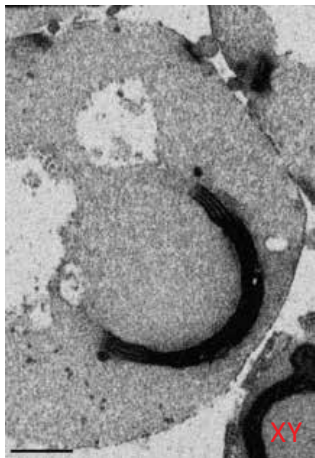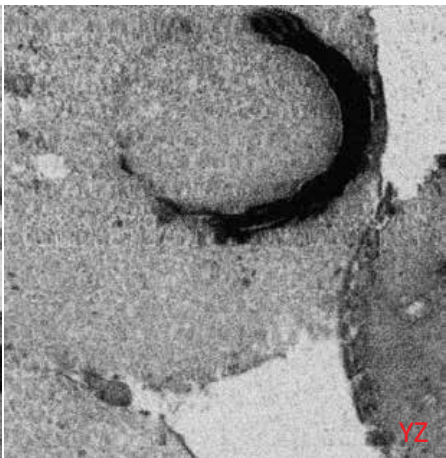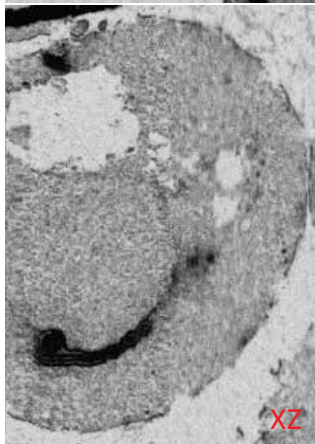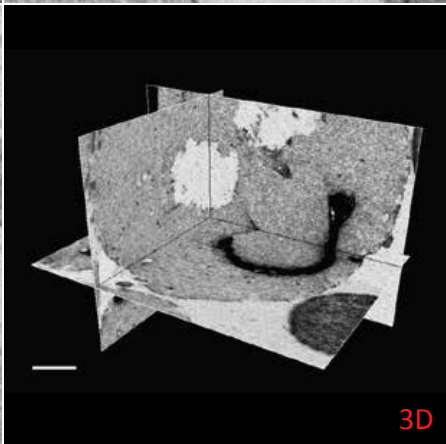**B**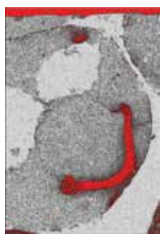**D**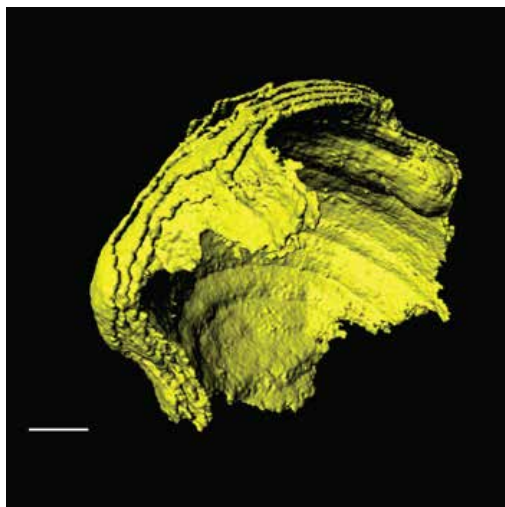**C**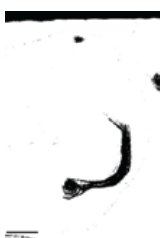**E**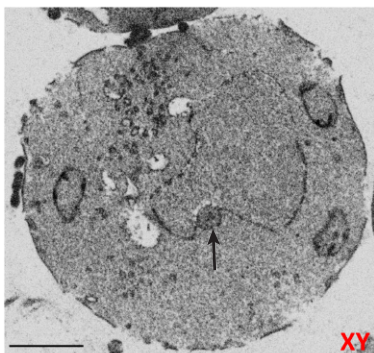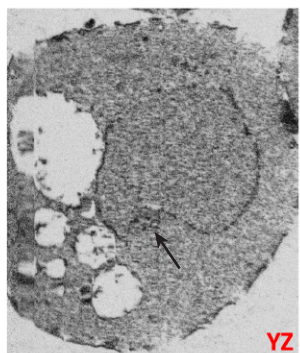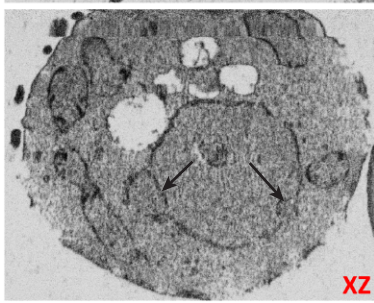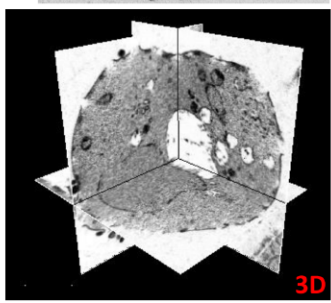

Supplement: FIG S1 [file mSphere.00981-19-sf001.pdf]
